# Hereditary Hemorrhagic Telangiectasia with Hepatic Vascular Malformations

**DOI:** 10.1155/2015/917818

**Published:** 2015-05-21

**Authors:** Yujiro Nishioka, Nobuhisa Akamatsu, Yasuhiko Sugawara, Junichi Kaneko, Junichi Arita, Yoshihiro Sakamoto, Kiyoshi Hasegawa, Norihiro Kokudo

**Affiliations:** Hepato-Biliary-Pancreatic Surgery Division, Department of Surgery, Graduate School of Medicine, The University of Tokyo, 7-3-1 Hongo, Bunkyo-ku, Tokyo 113-8655, Japan

## Abstract

Hereditary hemorrhagic telangiectasia (HHT) is a rare autosomal dominant hereditary disease. Early diagnosis is important to avoid complications from vascular lesions, but diagnosis is difficult in asymptomatic patients. A 69-year-old Japanese male patient was referred to our hospital for evaluation of hepatic vascular malformations. He had mild anemia with iron deficiency, and dynamic contrast-enhanced computed tomography revealed significant arteriovenous and arterioportal shunts throughout the liver. Telangiectasia from the pharynx to the duodenum was confirmed by gastrointestinal endoscopy. The patient history revealed episodes of epistaxis as well as a family history of epistaxis. He was diagnosed with HHT, although no other family member had been diagnosed with definite HHT. A diagnosis of HHT must be considered in patients with hepatic vascular malformations.

## 1. Background

Hereditary hemorrhagic telangiectasia (HHT), known as Osler-Rendu-Weber disease, is a rare autosomal dominant hereditary disease with an estimated prevalence of 1 in 5000 to 8000 [1, 2]. Patients with HHT usually present with symptoms related to vascular lesions in many organs: repeated epistaxis, gastrointestinal bleeding, and intracranial bleeding. The clinical diagnosis of HHT is based on the Curaçao criteria [3], and HHT is considered “probable” when two of the following criteria are present and “definite” when three or four criteria are present: epistaxis, telangiectasia, visceral vascular malformations, and a first degree relative with HHT. Although the international guideline indicates that early diagnosis of HHT is important to avoid complications from vascular lesions [4], diagnosis is difficult in asymptomatic patients with HHT.

Here we report a case of an asymptomatic patient in whom accidental discovery of HVMs eventually led to a diagnosis of definite HHT.

## 2. Case Presentation

A 69-year-old Japanese patient presented at a nearby hospital with low grade fever. Contrast-enhanced computed tomography (CT) scanning was performed to explore the cause of the disease, which incidentally revealed significant HVMs. He was subsequently referred to our institution for further examination.

Upon physical examination, he was afebrile and all other physical findings were in the normal range. He had no jaundice. He had a past medical history of only hypertension and had never had an episode of abdominal pain or difficulty in breathing. He had no history of smoking or alcohol abuse.

Laboratory tests revealed the following: white blood cell count 5700/mm^3^, hemoglobin 9.2 g/dL, mean corpuscular volume 79.9 fl, platelets 357,000/mm^3^, prothrombin time, international normalized ratio 0.96, albumin 4.2 g/dL, aspartate transaminase 21 IU/L, alanine transaminase 16 IU/L, alkaline phosphatase 262 IU/L, gamma-glutamyl transferase 72 IU/L, total bilirubin 0.7 mg/dL, serum iron 31 *μ*g/dL (normal range 64–187), unsaturated iron binding capacity 310 *μ*g/dL (normal range 126–358), and serum ferritin 19 ng/mL (normal range 13–277). Viral serologies for hepatitis B and C were both negative.

**Figure 1:**
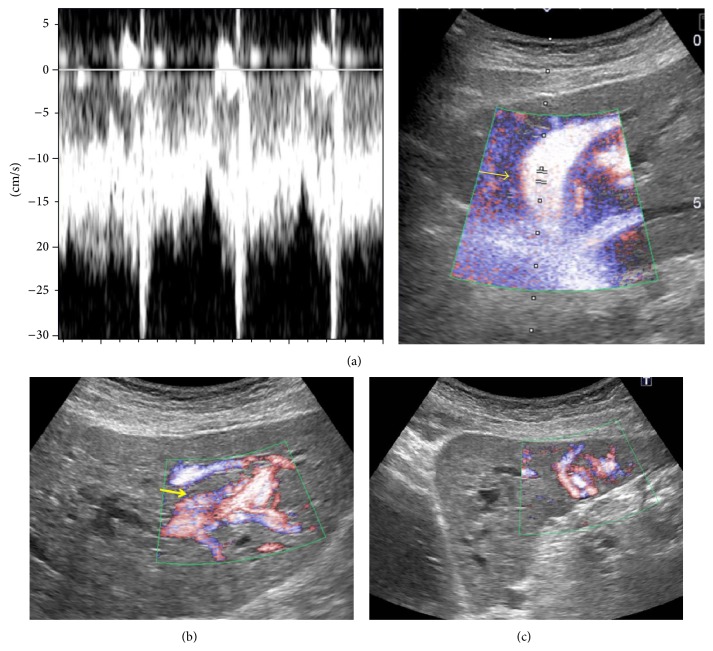
Abdominal ultrasonography with Doppler: (a) significantly dilated hepatic veins (arrow), in which not only venous but also arterial waves are demonstrated, (b) dilated hepatic arteries (arrow), and (c) peripheral vascular malformations.

Abdominal Doppler ultrasonography revealed HVMs with significantly dilated hepatic arteries and veins throughout the liver (Figure 1). The portal flow was hepatopetal, and there was no sign of cholecystitis.

**Figure 2:**
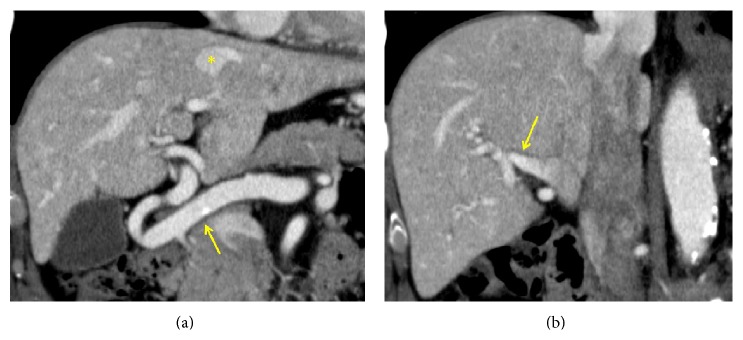
The arterial phase of dynamic contrast-enhanced CT scan: (a) significantly dilated extrahepatic artery (arrow), early enhancement of the hepatic veins (*∗*), (b) early enhancement of the right branch of the portal vein (arrow).

Dynamic contrast-enhanced CT revealed significantly dilated extrahepatic arteries and early enhancement of the hepatic veins and the right branch of the portal vein in the arterial phase (Figure 2). There was no splenomegaly. No vessel malformation was observed in the thoracic cavity.

An upper gastrointestinal endoscopy revealed telangiectasia from the pharynx to duodenum and on the tongue and the palate. Colonoscopy, however, revealed no telangiectasia or malignancy.

**Figure 3:**
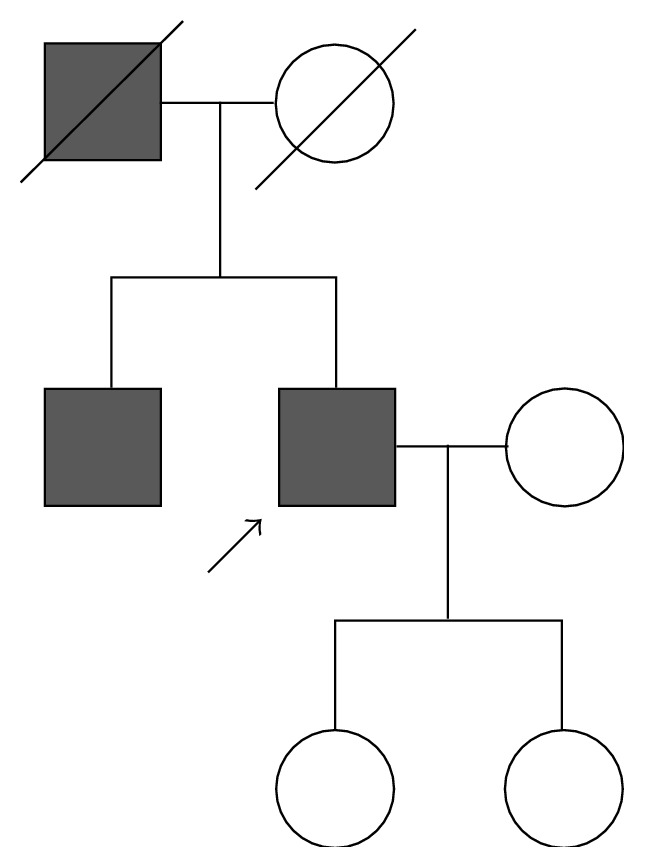
Pedigree of the patient's family. Arrow indicates the patient and gray box indicates those with repeated epistaxis.

In a detailed interview of the patient regarding his present illness and family history, he acknowledged frequent episodes of epistaxis for several years, and his father and brother had the same symptom (Figure 3). His mother and daughter had no episodes of epistaxis. Finally, the patient was diagnosed with definite HHT because he satisfied three of four factors of the Curaçao criteria.

Although transthoracic echocardiography revealed mild regurgitation through the tricuspid valve, there was no dilation of the right ventricle and the estimated systolic pressure of right ventricle (eRVSP) was 30 mmHg, which did not suggest a diagnosis of pulmonary hypertension (defined as an eRVSP value of over 50 mmHg). Moreover, there was no sign of right-sided heart failure.

Magnetic resonance angiography revealed no cerebral arteriovenous malformation.

To further elucidate the hepatic circulation and hemodynamics, hepatic angiography from the celiac artery and superior mesenteric artery was performed to directly visualize the arterioportal and arteriovenous shunt. Subsequent assessment of venous pressure with venography via a transjugular approach revealed that the inferior vena cava and the wedged pressure of the left hepatic vein were 20 mmHg and 25 mmHg, respectively. The hepatic-venous pressure gradient, namely, the subtraction of the inferior vena cava pressure from the wedged hepatic-venous pressure, was 5 mmHg, confirming the absence of portal hypertension, defined as a hepatic-venous pressure gradient value over 6 mmHg [5].

Finally, we proposed an analysis of the genotypic mutation, but the patient refused to undergo the analysis. Based on the examinations presented above indicating an asymptomatic patient, free from right-sided heart failure and liver dysfunction, we decided to follow him up with regular laboratory testing and transthoracic echocardiography at an outpatient clinic with oral administration of an iron preparation for the anemia.

## 3. Discussion

We describe a case of asymptomatic HHT presenting with prominent HVMs. HVMs are described in 41% to 84% of patients with HHT [6, 7], with several types of intrahepatic vascular shunts, such as arteriovenous shunts, arterioportal shunts, and portovenous shunts. High-output heart failure, portal hypertension, and ischemic biliary disease are the most common manifestations, but these are observed in only 8% of patients with HVMs associated with HHT [8]. In the present case, CT scanning of the patient accidentally revealed arteriovenous and arterioportal shunts, leading to a diagnosis of HHT. The patient actually had episodes of frequent epistaxis, but the degree of illness was too mild to be recognized as a disease.

Three of the four Curaçao criteria [3] were satisfied in the current case, recurrent and spontaneous epistaxis, telangiectasia in the oral cavity, and HVMs, leading to the diagnosis of “definite” HHT. The pathogenesis of HHT is heterozygous mutation in one of two genes, endoglin (ENG) or activin receptor-like kinase type 1 (ALK-1), which are both associated with the transforming growth factor *β* (TGF-*β*) superfamily signaling pathway expressed mostly in the vascular endothelium [8, 9]. The mutation of the ENG gene correlates with a severe and early emergence of symptoms and more frequent pulmonary and cerebral vascular malformations, referred to as an HHT-1 phenotypic pattern. On the other hand, the ALK-1 mutation correlates with a later onset and more frequent HVMs, referred to as an HHT-2 phenotypic pattern [10–12]. Patients with either HHT-1 or HHT-2 phenotypic pattern have epistaxis as the typical manifestation [12].

Fortunately, the present patient had no pulmonary or cerebral arteriovenous malformation, but screening for these malformations in patients with HHT is mandatory [4], and it is similarly important to confirm whether or not asymptomatic patients with vascular malformations have HHT, or those with HHT have vascular malformations. In patients with HVMs, a detailed interview regarding repeated epistaxis and the family history is necessary for the clinical diagnosis of HHT. It is essential for us to keep HHT in mind when patients with HVMs are encountered.

As for the mild anemia with iron deficiency in the current case, chronic oozing from the telangiectasia of the upper gastrointestinal tract could be a possible cause. In such cases, oral iron supplementation is recommended as first-line therapy [4] and was successful in the present case. Singh et al. [13] reported that four clinical factors are associated with the development of critical liver disease in patients with HHT: older age, female sex, lower hemoglobin levels, and elevated serum alkaline phosphatase levels. Additionally, they advocated a simple clinical scoring index using these four factors to estimate the probability of clinically significant liver disease. Based on the index, the present case was determined to have an “intermediate” risk and was expected to improve provided that his hemoglobin levels were adequately increased.

Liver transplantation is reported to be an effective treatment for symptomatic liver disease in HHT [14] and bevacizumab might be an alternative treatment [15]. In asymptomatic HHT patients, it is crucial to avoid the development of liver dysfunction, and close follow-up is mandatory.

## 4. Conclusions

We describe the case of a 69-year-old Japanese male patient with asymptomatic HVMs who was eventually diagnosed with HHT. HHT must be considered in patients with asymptomatic HVMs and a careful and detailed family history should be obtained.

## Abbreviations

HHT: Hereditary hemorrhagic telangiectasia
HVM: Hepatic vascular malformation
CT: Computed tomography
eRVSP: Estimated systolic pressure of the right ventricle
HVPG: Hepatic-venous pressure gradient.
